# PEX16: a multifaceted regulator of peroxisome biogenesis

**DOI:** 10.3389/fphys.2013.00241

**Published:** 2013-09-03

**Authors:** Peter K. Kim, Robert T. Mullen

**Affiliations:** ^1^Cell Biology Program, Department of Biochemistry, Hospital for Sick Children, University of TorontoToronto, ON, Canada; ^2^Department of Biochemistry, University of TorontoToronto, ON, Canada; ^3^Department of Molecular and Cellular Biology, University of GuelphGuelph, ON, Canada

**Keywords:** endoplasmic reticulum, organelle biogenesis, peroxin, peroxisome, PEX16

## Abstract

Peroxisomes are formed by two distinct pathways: the growth and fission of mature peroxisomes and *de novo* synthesis at the endoplasmic reticulum (ER). While many of the molecular mechanisms underlying these two pathways remain to be elucidated, it is generally accepted that their relative contribution to peroxisome formation may vary depending on the species, cell type and/or physiological status of the organism. One pertinent example of the apparent differences in the regulation of peroxisome biogenesis among evolutionarily diverse species is the involvement of the peroxin PEX16. In *Yarrowia lipolytica*, for instance, PEX16 is an intraperoxisomal peripheral membrane protein that participates in peroxisomal fission. By contrast, Human PEX16 is an integral membrane protein that is thought to function at the ER during the early stages of *de novo* peroxisome formation and also recruits peroxisomal membrane proteins directly to mature peroxisomes. Similarly, PEX16 in the plant *Arabidopsis thaliana* is speculated to be a PMP receptor at the ER and peroxisomes, and is also required for the formation of other ER-derived organelles, such as oil and protein bodies. Here we briefly review the current knowledge of *Y. lipolytica,* human and *A. thaliana* PEX16 in the context of our overall understanding of peroxisome biogenesis and the role of the ER in this process in these three divergent species.

## Introduction

Peroxisomes are found in virtually all eukaryotic organisms and while they possess a somewhat simple architecture consisting of a nonhomogenous matrix enclosed by a single membrane, their metabolic functions are highly complex (Islinger et al., 2010). For instance, peroxisomes in plants participate in a remarkable array of processes, including the glyoxylate cycle and the synthesis of phytohormones (Hu et al., 2012), while in humans the organelle is involved in cholesterol and bile acid biosynthesis, and defects in the organelle result in a number of lethal genetic disorders (Waterham and Ebberink, 2012). In yeasts, peroxisomes are required for metabolizing nonfermentable carbon sources such as methanol and oleate (Van Der Klei and Veenhuis, 2006). Notably, this metabolic feature has been readily exploited for the identification of yeast mutants with defects in the biogenesis of peroxisomes and the subsequent identification of the corresponding genes and their protein product (collectively referred to as peroxins or PEX proteins) [reviewed in Distel et al. (1996); see also Tower et al. (2011)].

To date, over 30 peroxins involved in the key steps underlying peroxisome biogenesis in yeast have been identified, many of which are also present in other eukaryotes, including mammals and plants (Hayashi and Nishimura, 2006; Kiel et al., 2006). Pertinent examples of these conserved peroxins include those involved in peroxisomal matrix protein import (PEX5, 7, 10, 12, 13, etc.) and those that help orchestrate the growth and division of peroxisomes (e.g., PEX11 protein family). For more detailed information regarding these processes and the peroxins involved, we refer the reader to several recent reviews (Hu, 2010; Koch and Brocard, 2011; Liu et al., 2012; Schrader et al., 2012).

PEX3, PEX16, and PEX19 are another important set of peroxins that are generally referred to as “early” peroxins because of their essential roles in the initial steps of peroxisome biogenesis (Schliebs and Kunau, 2004). However, the precise roles of these peroxins appear to vary considerably depending on the organism. For instance, PEX19 serves in all peroxisome-containing species as a soluble receptor for nascent peroxisomal membrane proteins (PMPs) by binding and targeting them to the peroxisomal membrane (Ma et al., 2011; Theodoulou et al., 2013), but, in the yeast *Saccharomyces cerevisiae*, PEX19 functions also in peroxisome inheritance (Otzen et al., 2012). Likewise, PEX3 is a conserved membrane-bound docking receptor for incoming complexes of PEX19 and its PMP cargo (Sato et al., 2010; Schmidt et al., 2010), yet yeast PEX3 serves also in peroxisome inheritance and in the degradation of peroxisomes (Munck et al., 2009; Motley et al., 2012; Nordgren et al., 2013). PEX16 seems to possess the most diverse set of functions, ranging from a matrix-localized, peripheral membrane protein involved in peroxisomal fission in the yeast *Y. lipolytica* (Guo et al., 2007), to an integral membrane-bound PMP receptor at the ER and peroxisomes in mammals (Kim et al., 2006; Matsuzaki and Fujiki, 2008), and perhaps also in plants (Karnik and Trelease, 2007). Notably, PEX16 homologs are absent in some well characterized model organisms, including *S. cerevisiae* (Kiel et al., 2006) and *Caenorhabditis elegans* (Thieringer et al., 2003).

**Figure 1:**
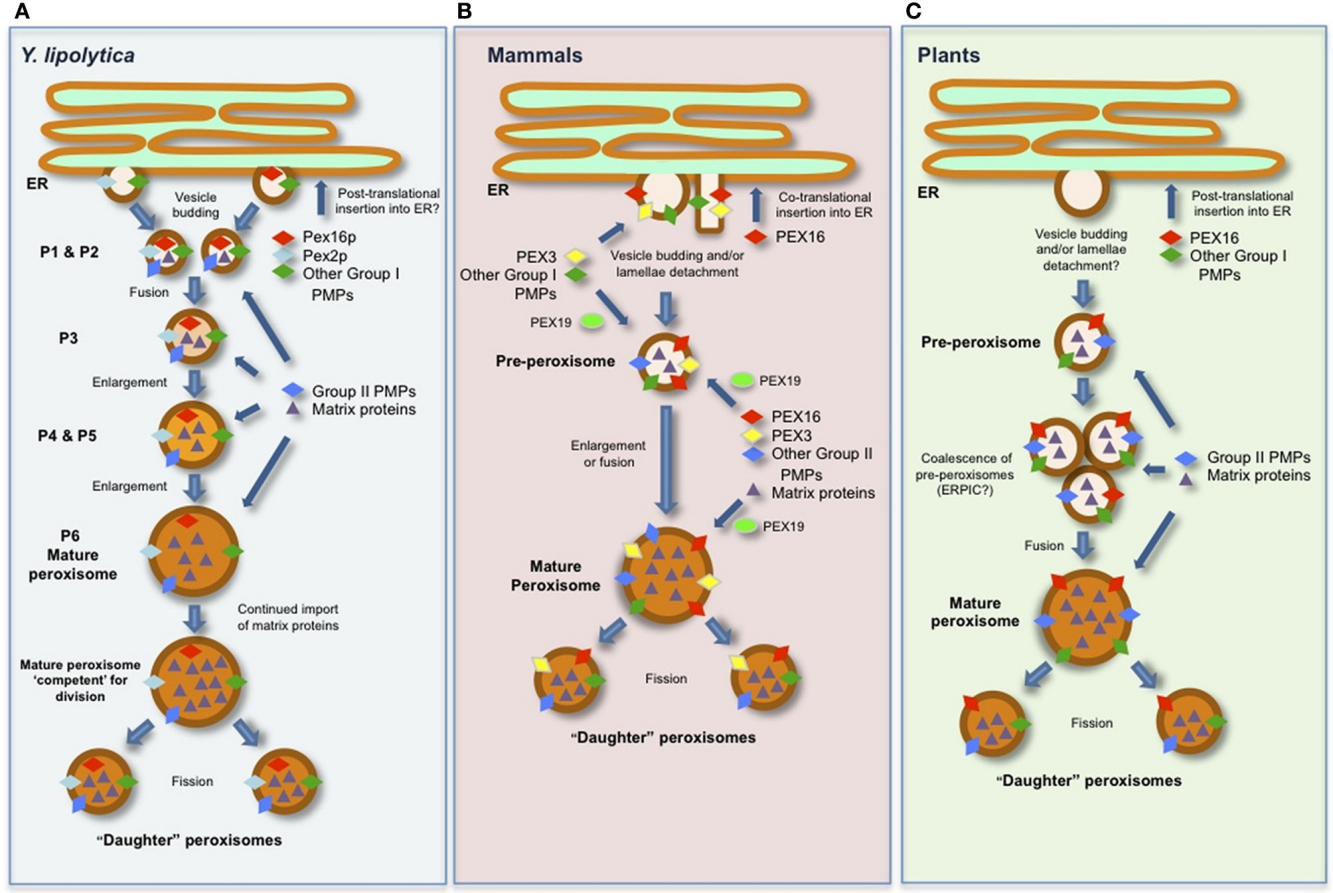
**Schematic representations of generalized models for the biogenesis of peroxisomes and the role(s) of PEX16 in **(A)***Y. lipolytica*, **(B)** mammals (human), and **(C)** plants (*Arabidopsis*).** See text for details and references.

Interestingly, the results obtained from studies of PEX16 have been instrumental in the development of our current working models for peroxisome biogenesis, and have shed significant light on the role that ER plays in this process in evolutionarily distinct organisms (Figure 1) (Titorenko and Rachubinski, 2009; Hu et al., 2012; Dimitrov et al., 2013; Tabak et al., 2013). There is also a growing appreciation that there are differences in the relative contribution of these two pathways, as well as their underlying molecular mechanisms, to the biogenesis of peroxisomes in different organisms (Koch and Brocard, 2011; Islinger et al., 2012). Thus, it is not always appropriate to extrapolate the knowledge gained from one organism to another, and a unified model of peroxisome biogenesis, for either pathway, may not be feasible.

Here we briefly highlight the functional properties and intracellular trafficking pathways of PEX16 from the three species wherein this peroxin has been the best studied—*Y. lipolytica*, human, and *A. thaliana*—and, in doing so, how this knowledge has been incorporated into the models for peroxisome biogenesis among these evolutionarily diverse species.

## *Yarrowia lipolytica* PEX16P

**Figure 2:**
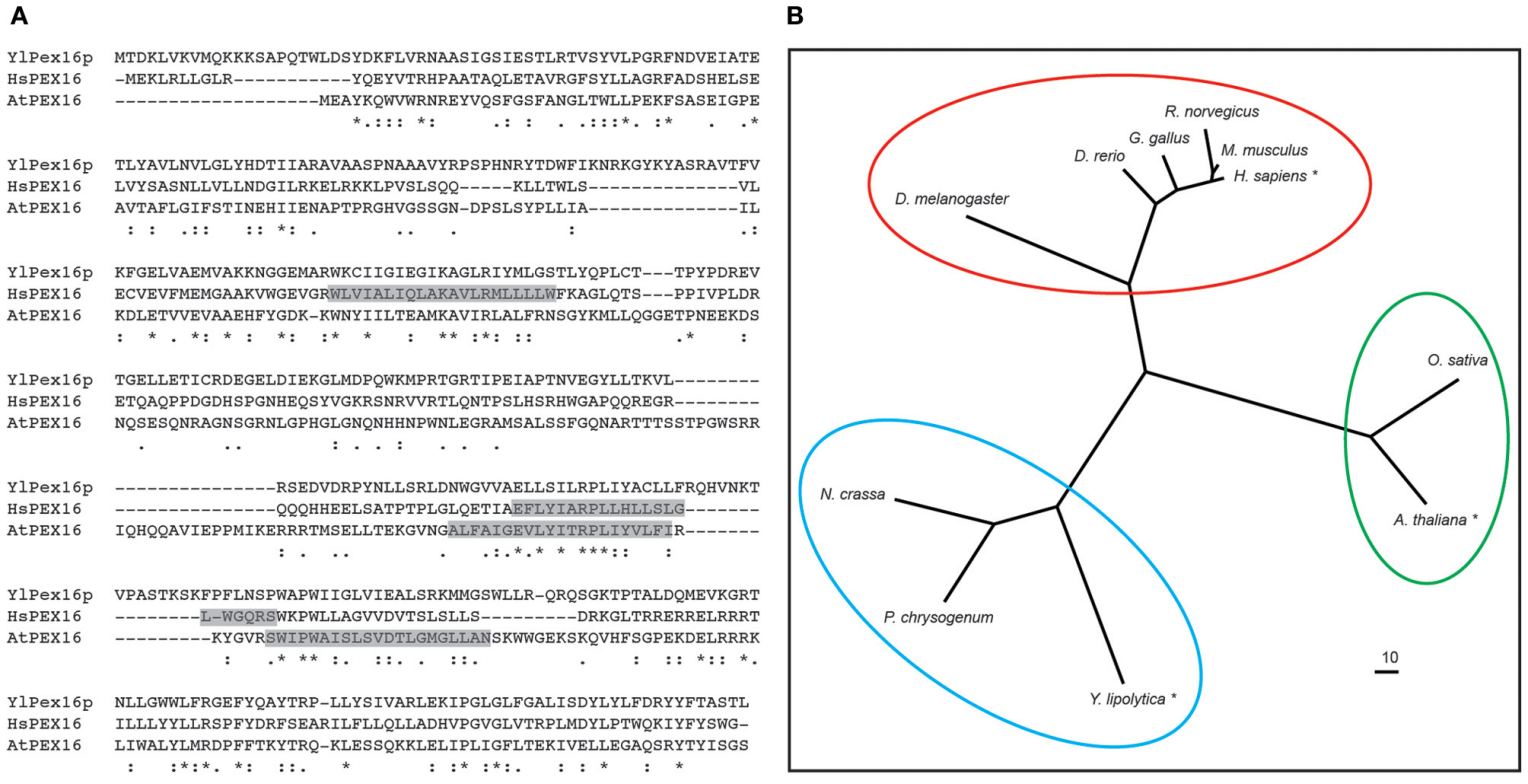
**Polypeptide sequence alignment and phylogenetic analysis of various PEX16 proteins. (A)** Deduced amino acid sequence alignment of *Y. lipolytica* Pex16p (YlPex16p), human (*Homo sapiens*) PEX16 (HsPEX16), and *A. thaliana* PEX16 (AtPEX16). Identical residues are indicated with asterisks, strongly and weakly similar residues are indicated with a colons and periods, respectively. Predicted membrane-spanning sequences in HsPEX16 and AtPEX16 are shaded and based on Honsho et al. (2002) and Karnik and Trelease (2007), respectively. **(B)** Phylogenetic analysis of PEX16 sequences from selected evolutionarily diverse species. Each protein is labeled based on its respective Genus and species, and those shown in **(A)** are indicated with an asterisk, and circles represent PEX16 proteins of the metazons, yeasts (fungi), and plants that form distinct clades. Branch lengths of the tree are proportional to divergence with the “10” scale bar representing a 10% change. Sequence alignments were carried out using either CLUSTALW (Larkin et al., 2007) and the phlyogram was generated using the program TreeView (v1.6.6). Genbank^®^ accession numbers are as follows: *H. sapiens* (BAA88826.1), *Rattus norvegicus* (NP_001012088.1), *Mus musculus* (NP_660104.2), *Drosophila melanogaster* (NP_649252.1), *Neurospora crassa* (XP_963884.2), *Danio rerio* (NP_001020340.1), *Gallus gallus* (XP_421125.3), *Penicillium chrysogenum* (ABH11422.1), *Y. lipolytica* (AAB41724.1), *A. thaliana* (NP_566053.1), *Oryza sativa* (EEC72380.1).

The PEX16 protein was first described in *Y. lipolytica* (Eitzen et al., 1997). In this study, a *Ylpex16* mutant strain was identified based on its inability to use oleate as a sole carbon source and subsequent cloning of the *YlPEX16* gene revealed it encoded a protein that had no obvious structural/functional domains and no significant sequence homology with any other functionally characterized protein. Phylogenetic analysis of sequences present in extant genome databases, however, reveals that PEX16 homologs exist in most, but not all, eukaryotes and that they share approximately 15–25% sequence identity (Figure 2A). PEX16 homologs from metazoans, yeasts and plants are also separated into distinct clades (Figure 2B), indicating early diversification and perhaps functional specialization.

The initial study of YlPex16p revealed that the protein is peripherally associated with the inner surface of the peroxisomal membrane (Eitzen et al., 1997) and that overexpression of *YlPEX16* yielded a reduced number of larger peroxisomes compared to those in wild-type cells, revealing that YlPex16p is required for peroxisomal fission. Additional studies on YlPex16p, as well as other studies aimed at deciphering how peroxisomes are formed and maintained in *Y. lipolytica,* have since led to the development of a sophisticated model of peroxisome biogenesis in this organism where YlPex16p plays a critical role in peroxisome division (Titorenko and Rachubinski, 2001; Boukh-Viner and Titorenko, 2006) As depicted in Figure 1A, this model includes six distinct peroxisomal subcompartments, termed P1–P6, which are organized into a multi-step biogenetic pathway. The earliest of these subcompartments, the so-called pre-peroxisomes P1 and P2, are considered to bud as vesicles from a specialized region of the ER and contain a unique subset of PMPs, including YlPex16p, which are collectively known as group I PMPs, i.e., PMPs that sort initially to the ER and then to peroxisomes. Thereafter, P1 and P2 are thought to fuse to form the P3 subcompartment, which in turn enlarges due to the continual import of matrix proteins and/or group II PMPs directly from the cytosol to form P4, then P5, and eventually a mature peroxisome (P6), which can subsequently divide into new “daughter” peroxisomes.

YlPex16p is thought to function by binding the membrane lipid lyso-phosphatidic acid (LPA) in the matrix-facing leaflet of the P1–P5 membranes (Figure 1A), thereby inhibiting fission of the P1–P5 subcompartments by suppressing the synthesis of LPA-derived diacylglyercol (DAG), a unique cone-shaped lipid that induces membrane curvature (Guo et al., 2003). In a mature (P6) peroxisome, however, the continued import of nascent matrix proteins eventually results in the organelle being “overloaded” with matrix protein constituents and, thus, competent for division (Figure 1A) (Guo et al., 2007). At this point, the enzyme acyl-CoA oxidase is thought to relocalize from the matrix to the membrane where it binds to YlPex16p and stimulates a decrease in its affinity for LPA. This leads in turn to an increase in the formation of DAG from LPA, which, along with phosphatidylserine (PS), “flips” between the leaflets of the peroxisomal membrane bilayer, causing lipid asymmetry that leads to bending of the membrane and the subsequent division of the organelle upon the recruitment of the peroxisome division machinery (Guo et al., 2007).

Besides its unique role in peroxisome division, YlPex16p is perhaps best known as one of the first PMPs experimentally shown to target indirectly to peroxisomes via the ER (Titorenko and Rachubinski, 1998), and thus, early evidence for a role of the ER in formation and maintenance of peroxisomes. However, the nature of the molecular machinery and targeting signals responsible for the ER-to-peroxisome sorting of YlPex16p are unknown.

## Human PEX16

Unlike YlPex16p, human PEX16 (HsPEX16) is an integral membrane protein containing at least two transmembrane domains (TMDs) (Figure 2A) and a topological orientation whereby both the N and C termini face the cytosol (Honsho et al., 2002). The *Y. lipolytica* and human proteins also differ in that YlPex16p presumably inserts into the ER in a post-translational manner, as do all PMPs that sort to peroxisomes via the ER in *S. cerevisiae* (Van Der Zand et al., 2010), while the insertion of HsPEX16 into the ER occurs in a co-translational manner (Kim et al., 2006). HsPEX16 is also distinct from YlPex16p in that it does not appear to be directly involved in regulating peroxisome division, but, instead, functions as a PMP receptor during the early stages of *de novo* peroxisome formation at the ER, as well as in mature peroxisomes (Kim et al., 2006; Matsuzaki and Fujiki, 2008). Consistent with this, the loss of HsPEX16, unlike YlPex16p, results in the complete absence of any peroxisomal structures (Honsho et al., 2002).

The precise role of HsPEX16 during the *de novo* synthesis of peroxisomes seems to be as a receptor responsible for the integration of the peroxin PEX3 into the ER and, thus, possibly the subsequent insertion of other PEX3-dependent group I PMPs at the ER (Fransen et al., 2001; Kim et al., 2006; Matsuzaki and Fujiki, 2008). As mentioned previously, most nascent PMPs in the cytosol are recognized and bound by PEX19, a soluble receptor/chaperone that delivers its PMP “cargo” to the membrane-bound docking receptor PEX3. While the details of how the PEX3 receptor mediates the integration and assembly of PMPs into membranes are largely unknown, the way in which PEX3 itself is inserted into membranes seems to also vary depending on the organism. For instance, in *S. cerevisiae*, which lacks a Pex16p homolog, Pex3p is inserted post-translationally into ER membranes via the SEC61 complex (Van Der Zand et al., 2010; Thoms et al., 2012). In mammals, however, the import of PEX3 does not appear to rely on SEC61 (South et al., 2000), but does rely on HsPEX16 (Kim et al., 2006).

Based on these and other findings (Huybrechts et al., 2009), the working model for peroxisome biogenesis in mammals (Figure 1B) includes HsPEX16 serving as the receptor for PEX19-independent insertion of PEX3 at the ER. Thereafter, PEX16, PEX3, and the group I PMPs, which are subsequently recruited to the ER by PEX3, are incorporated into a pre-peroxisome in a process that appears to require the ER export factor SEC16B (Yonekawa et al., 2011). The structure of a pre-peroxisome in mammals, however, appears to vary, since some evidence suggests they are small vesicles, as in yeasts, while in certain specialized cells they resemble a lamellar extension that detaches *en block* from the ER (Geuze et al., 2003). Regardless, pre-peroxisomes in mammals are considered to be competent for nascent PMP import (i.e., group II PMPs) and matrix proteins from the cytosol to either enlarge into a new mature peroxisome or fuse with a pre-existing mature (or “daughter”) peroxisome in order to promote its growth (Figure 1B) (Kim et al., 2006).

Similar to its role at the ER, HsPEX16 appears to function as a receptor for PEX3 in mature peroxisomes as well, and, in doing so, facilitating the subsequent PEX3-dependent import of group II PMPs into more mature organelles (Matsuzaki and Fujiki, 2008) (Figure 1B). However, the molecular mechanism underlying the import of PEX3 by PEX16 at peroxisomes seems to be distinct from that at the ER (Kim et al., 2006), since it is dependent on PEX19 (Matsuzaki and Fujiki, 2008). Conversely, HsPEX16 also targets directly to peroxisomes and does so in a post-translational, PEX3- and PEX19-dependent manner (Matsuzaki and Fujiki, 2008). These findings, and those discussed above for the role of HsPEX16 as a receptor for PEX3 at both the ER and peroxisomes, has led to the suggestion of a “chicken-or-the-egg” dilemma for how these two PMP receptors operate in a spatiotemporal manner (Matsuzaki and Fujiki, 2008). However, given that HsPEX16 is inserted into the ER via the SEC61 co-translational import pathway and that PEX3 is only found at the ER in the presence of HsPEX16 (Kim et al., 2006), it seems that HsPEX16 acts as the “master” peroxin responsible for the initiation of peroxisome biogenesis at the ER in mammals.

## *Arabidopsis thaliana* PEX16

While the *de novo* synthesis of peroxisomes is a possibility in plants, there is almost no direct evidence in support of this pathway. Rather, the role of the ER in peroxisome biogenesis in plants is thought to serve strictly as the site from which group I PMPs and phospholipids are trafficked (via pre-peroxisomes) to mature peroxisomes (Figure 1C). For a more comprehensive discussion on the role of the ER in plant peroxisome biogenesis refer to Trelease and Lingard (2006) and Hu et al. (2012).

Among the plant PMPs that sort to peroxisomes via the ER is *Arabidopsis* PEX16, a membrane protein that, like HsPEX16, possesses two predicted TMDs (Figure 2A) (Karnik and Trelease, 2007). AtPEX16 was initially identified in a study of the *shrunken seed 1* (*sse1*) mutant in *Arabidopsis*. Herein, SSE1 was re-annotated as PEX16 based on its sequence similarity to *Yl*Pex16p (Figure 2A) and its ability to complement, albeit partially, the *Ylpex16* mutant (Lin et al., 1999). While the latter observation implies that AtPEX16 and YlPex16p operate in a similar manner during peroxisome division, which is supported by the observation that *Atpex16* knockdown cells possess fewer and enlarged peroxisomes (Nito et al., 2007), similar to *Ylpex16* mutant yeast cells, other studies indicate that AtPEX16 plays additional roles during plant peroxisome biogenesis. For instance, similar to *Hspex16* mutant cells, the *Atpex16* null mutant is devoid of normal peroxisomes (Lin et al., 2004), implying that AtPEX16 functions at the early stages of peroxisome biogenesis. Whether the *AtPEX16* gene can functionally complement the *Hspex16* mutant, or vice versa, has not been reported.

The intracellular localization and trafficking mechanisms of AtPEX16, including its molecular targeting signals, have been relatively well studied (Karnik and Trelease, 2007). Overall, these findings have not only helped to develop ideas on the possible roles of AtPEX16 in peroxisome biogenesis, but have also helped formulate the larger models for overall peroxisome biogenesis in plants, particularly for how the ER participates in this process (Figure 1C). For instance, that AtPEX16 localizes to both the ER and peroxisomes or to peroxisomes only depending on the tissue/cell type (Lin et al., 2004; Karnik and Trelease, 2005) supports the idea that this protein serves more than one function in the plant peroxisome biogenetic pathway, e.g., at the ER, AtPEX16, like HsPEX16, may act as a PMP receptor and help orchestrate the sorting of these PMPs into pre-peroxisomes. Notably, *Atpex16* mutant plants have defects not only in peroxisomal biogenesis, but also in the formation of other ER-derived organelles, such as oil and protein bodies (Lin et al., 1999), suggesting that the roles of PEX16 at the ER in plants may actually extend beyond those ascribed to its mammalian counterpart.

As depicted also in Figure 1C, the localization of AtPEX16 at mature peroxisomes and perhaps at pre-peroxisomes enroute to peroxisomes has been attributed to the protein's potential role as a receptor for PEX3 and other group II PMPs (Karnik and Trelease, 2007). It is not known, however, whether AtPEX16 can target directly to pre-peroxisomes and/or mature peroxisomes in a post-translational manner, similar to HsPEX16, although it does target post-translationally to the ER (Karnik and Trelease, 2007).

The ER-to-peroxisome trafficking relies on two sets of overlapping molecular targeting signals: (i) those responsible for directing the protein from its sites of synthesis in the cytosol to the ER and (ii) those that direct it from the ER to peroxisomes (Karnik and Trelease, 2007). While the precise nature of these signals in AtPEX16 is an open question, the trafficking of AtPEX16 from the ER to peroxisomes appears to involve a so-called ER-peroxisome-intermediate-compartment (ERPIC), which is postulated to comprise ER-derived pre-peroxisomes that have coalesced prior to their fusion with mature peroxisomes (Karnik and Trelease, 2007). ERPIC-like compartments have been also identified in certain yeast and mammals (Titorenko and Mullen, 2006), although in no case, including in plants, have these been thoroughly investigated.

## Conclusions and perspectives

One of the key regulators of peroxisome biogenesis is PEX16, a peroxin that, depending on the organism, functions in remarkably diverse ways, including the control of peroxisome fission [e.g., *Y. lipolytica* Pex16p (Guo et al., 2003, 2007)], or the de *novo* synthesis of peroxisomes [e.g., human PEX16 (Kim et al., 2006)]. On the other hand, it is equally remarkable that some organisms, such as *S. cerevisiae*, lack a PEX16 homolog (Kiel et al., 2006), yet their mode of peroxisome biogenesis is similar to Y. *lipolytica* (Van Der Zand et al., 2012), implying that they rely instead on an alternative mechanism(s), or other proteins that provide similar functions, for the control of key steps during peroxisome biogenesis. One possible explanation for this apparent loss of PEX16, at least in *S. cerevisiae*, is that all of the PMPs in this yeast are inserted into the ER via the SEC61 complex (Van Der Zand et al., 2010; Thoms et al., 2012). By contrast, in mammals, PEX16, not SEC61 (South et al., 2000), appears to mediate (presumably through PEX3) the insertion of PMPs that localize to peroxisomes via the ER (Kim et al., 2006), as well as PMPs that target directly to peroxisomes (Matsuzaki and Fujiki, 2008). Whether this premise holds true remains to be determined. Regardless, how PEX16 actually functions as a PMP receptor at the ER and peroxisomes, perhaps by forming part of a translocon analogous to the SEC61 complex, and how it regulates peroxisome division, which seems to rely on a dynamic interplay of peroxisomal proteins and lipids (Guo et al., 2007; Itoyama et al., 2012), will be fascinating subjects for future research. It is also conceivable that future cross complementation and heterologous expression studies between various yeast, mammalian, and plant species may reveal as-yet-unknown aspects of PEX16 in peroxisome biogenesis and, by doing so, will provide additional insight to the shared and/or unique spatiotemporal dynamics and molecular mechanisms that underlie the peroxisome biogenetic pathways in different organisms.

### Conflict of interest statement

The authors declare that the research was conducted in the absence of any commercial or financial relationships that could be construed as a potential conflict of interest.
